# Massachusetts Medical Society and the Homeopathists

**Published:** 1873-07

**Authors:** 


### Massachusetts Medical Society and the Homeopathists.

The legal right of the Massachusetts Medical Society to expel members practising Homeopathy has been sustained by the courts, and at its last meeting, after giving a detail of the trial of charges against the Homeopathic practitioners, the secretary of the society said: "Mr. President, I move the acceptance of the records and report of the Board of Trial, and that the following vote be passed:

*Voted.* Upon the report of the Board of Trial for the trial of the charges against William Bushnell, Milton Fuller, Herman L. H. Hoffendahl, George Russell, Israel T. Talbot, David Thayer and Benjamin H. West—that the said William Bushnell, Milton Fuller, Herman L. H. Hoffendahl, George Russell, Israel T. Talbot, David Thayer and Benjamin H. West *be and are* expelled from the Massachusetts Medical Society." The vote was then passed with only one dissenting vote, and the result was welcomed with a round of applause.

